# Expression of ANO1/DOG1 is associated with shorter survival and progression of breast carcinomas

**DOI:** 10.18632/oncotarget.23078

**Published:** 2017-12-09

**Authors:** Jun Sang Bae, Jeong Yeol Park, See-Hyoung Park, Sang Hoon Ha, Ae Ri An, Sang Jae Noh, Keun Sang Kwon, Sung Hoo Jung, Ho Sung Park, Myoung Jae Kang, Kyu Yun Jang

**Affiliations:** ^1^ Department of Pathology, Chonbuk National University Medical School, Research Institute of Clinical Medicine of Chonbuk National University-Biomedical Research Institute of Chonbuk National University Hospital and Research Institute for Endocrine Sciences, Jeonju, Republic of Korea; ^2^ Department of Forensic Medicine, Chonbuk National University Medical School, Research Institute of Clinical Medicine of Chonbuk National University-Biomedical Research Institute of Chonbuk National University Hospital and Research Institute for Endocrine Sciences, Jeonju, Republic of Korea; ^3^ Department of Bio and Chemical Engineering, Hongik University, Sejong, Republic of Korea; ^4^ Division of Biotechnology, Chonbuk National University, Iksan, Republic of Korea; ^5^ Department of Preventive Medicine, Chonbuk National University Medical School, Jeonju, Republic of Korea; ^6^ Department of Surgery, Chonbuk National University Medical School, Jeonju, Republic of Korea

**Keywords:** breast, carcinoma, ANO1, DOG1, prognosis

## Abstract

The expression of ANO1 is considered to have diagnostic specificity for gastrointestinal stromal tumors. However, its function as a calcium-activated chloride channel suggests that the expression of ANO1 is not restricted to gastrointestinal stromal tumors. Recently, it has been reported that ANO1 has roles in the progression of human malignant tumors. However, the role of ANO1 in breast carcinoma has been controversial. Therefore, we investigated the expression of ANO1 in 139 breast carcinoma patients and the role of ANO1 *in vitro*. The immunohistochemical expression of ANO1 was significantly associated with the expression of β-catenin, cyclin D1, MMP9, snail, and E-cadherin. Especially, ANO1 expression was an independent indicator of poor prognosis of shorter overall survival and relapse-free survival of breast carcinoma patients by multivariate analysis. In MCF7 and MDA-MB-231 breast carcinoma cells, inhibition of ANO1 with T16Ainh-A01 or siRNA for ANO1 significantly suppressed the proliferation of cells. Knock-down of ANO1 with siRNA induced G0/G1 cell cycle arrest and significantly inhibited the invasiveness of breast carcinoma cells. Knock-down of ANO1 decreased the expression of β-catenin, cyclin D1, MMP9, snail, and N-cadherin, and increased the expression of E-cadherin. In conclusion, this study demonstrates that ANO1 expression is an indicator of poor prognosis of breast carcinoma patients and suggests that ANO1 might be a therapeutic target for breast carcinoma patients with ANO1-positive tumors and poor prognosis.

## INTRODUCTION

ANO1 is a member of the TMEM16 family and characterized by its role as a calcium-activated chloride channel [1]. ANO1 is also known as DOG1, TMEM16A, ORAOV2 and TAOS2 [2]. In clinical oncology, the expression of ANO1/DOG1 was considered to have diagnostic specificity for determining which tumor is gastrointestinal stromal tumor to consider targeted therapy [3]. However, its biologic characteristic as a calcium-activated chloride channel [1, 4, 5] suggests that ANO1/DOG1 might be involved in various pathophysiologic statuses [2]. Supportively, the expression of ANO1 was not restricted to the interstitial cells of Cajal or gastrointestinal stromal tumors and was widely expressed in normal tissues, such as smooth muscle and epithelial cells [6], and various human tumors, such as renal oncocytoma, chromophobe renal cell carcinoma, pancreatic neoplasms, salivary gland neoplasms, synovial sarcoma, leiomyoma, pancreatic adenocarcinoma, and leiomyosarcoma [7]. The possibility that ANO1 might be involved in the tumorigenesis has been suggested by its chromosomal location at 11q13 because this loci is frequently amplified in various human malignant tumors, such as head and neck cancer, breast carcinoma (BCA), lung cancer, and esophageal cancer [8]. Furthermore, higher expression of ANO1 was observed in cancer tissue compared with normal counterpart tissue in gastric carcinomas [9], hepatocellular carcinoma [10], and prostatic carcinomas [11]. In glial tumors, higher expression of ANO1 was correlated with higher histologic grade of tumors [12]. In addition, higher expression of ANO1 was significantly associated with advanced phenotypes of prostatic carcinomas [11] and oral squamous cell carcinomas [13]. Moreover, the expression of ANO1 was significantly correlated with shorter survival of gastric carcinomas [9] and head and neck squamous cell carcinomas [14]. In tumorigenesis, it has been reported that ANO1 expression was associated with the proliferation, invasiveness, and apoptosis of cancer cells and inhibition of ANO1 suppressed the growth and progression of human cancer cells [10, 12, 15–17]. It has been suggested that ANO1 is involved in tumorigenesis by either its role as a calcium-dependent chloride channel or as a signaling molecules. Signaling pathways which may be involved in the progression of human cancers in association with ANO1 include the MAPK signaling [10, 17], cell cycle regulation pathway [10, 12, 17], the EGFR pathway [18], and epithelial-mesenchymal transition (EMT) [9, 12]. However, there are conflicting reports regarding the role of ANO1 in human malignant tumors. In some reports, loss of ANO1 was associated with increased EMT and lymph node metastasis [19]. In addition, the expression of ANO1 was not involved in the proliferation of cancer cells or the survival of cancer patients [14, 20, 21]. In some populations of BCAs, ANO1 expression was associated with favorable prognosis [22]. Therefore, further study is needed to clarify the exact role of ANO1 in human malignant tumors.

BCA is one of the most common malignant tumors in females and the second leading cause of cancer-related death in females [23]. In breast, it has been reported that ANO1 is endogenously expressed in normal breast [24] and previous analysis of public data revealed higher mRNA expression of ANO1 in BCA tissues compared with normal breast tissue [25]. In BCA cells, ANO1 induced proliferation of cells by activating EGFR signaling and inhibition of ANO1 reduced EGFR signaling [18, 26]. However, there was a report that the association between ANO1 expression and the prognosis of BCA patients is not clear [21], and some reports have shown that the expression of ANO1 predicts favorable prognosis in a subpopulation of BCA patients who have tumors which are progesterone receptor (PR)-positive or human epidermal growth factor receptor 2 (HER2)-negative [22, 27]. Therefore, the role of ANO1 in BCAs needs to be clarified. We investigated the prognostic significance of ANO1 expression in human BCA tissue samples and investigated the effects of ANO1 expression in BCA cells to clarify role of ANO1 in BCAs.

## RESULTS

### The expression of ANO1 is associated with shorter survival of breast carcinoma patients

**Figure 1:**
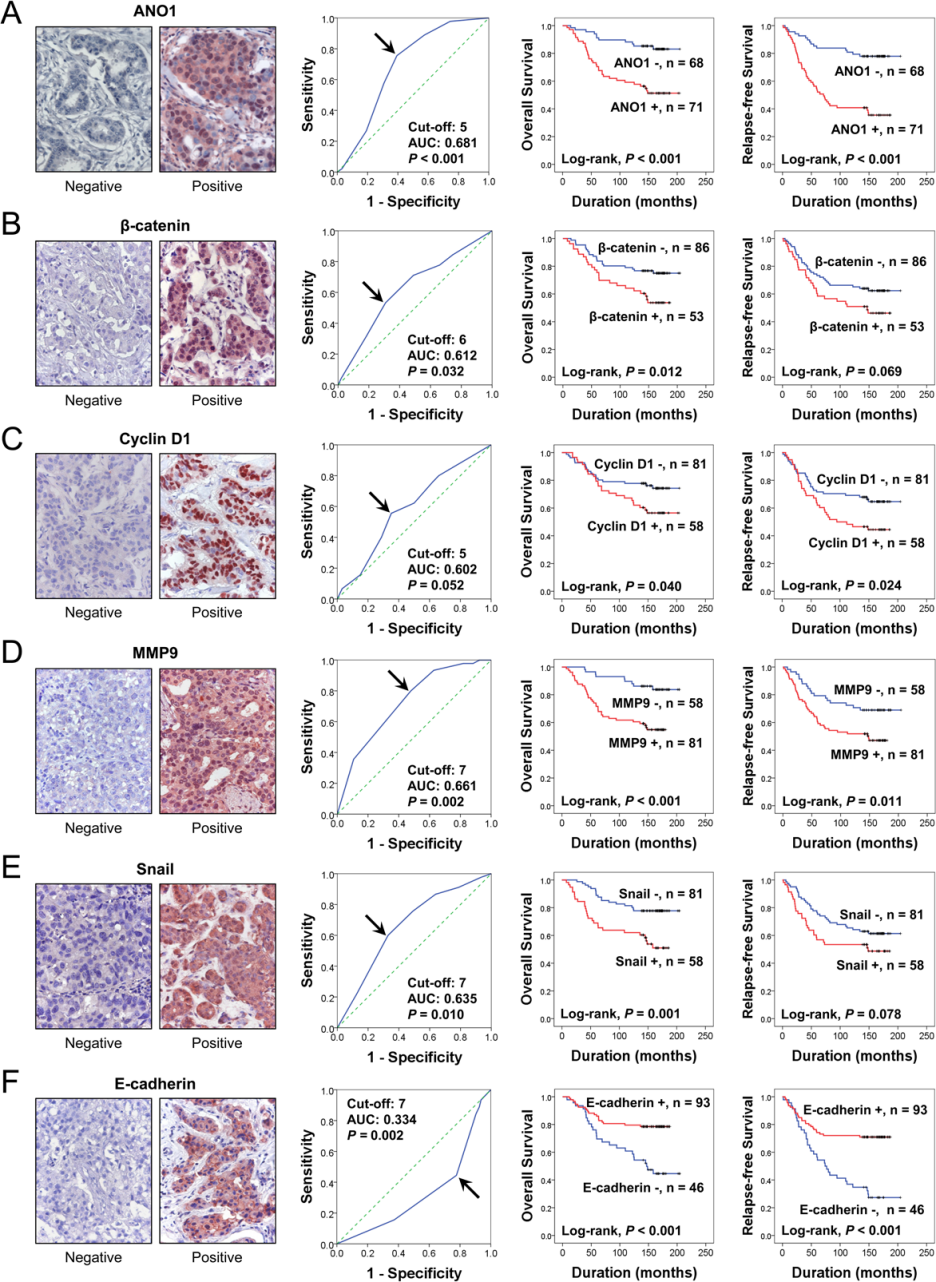
Expression and prognostic significance of ANO1, β-catenin, cyclin D1, MMP9, snail, and E-cadherin in 139 human breast carcinomas (**A–F**) Immunohistochemical expression of ANO1 (A), β-catenin (B), cyclin D1 (C), MMP9 (D), snail (E), and E-cadherin (F) in breast carcinoma. Receiver operating characteristic curve analysis to determine the cut-off point for the immunohistochemical staining and to determine the most predictive point for estimating the survival of breast carcinoma patients. The arrows indicate the cut-off points for each immunostaining. The survival curves for the overall survival and relapse-free survival were derived from Kaplan-Meier survival analysis. AUC; area under the curve. Original magnification of immunohistochemical images; ×400.

Immunohistochemical expression of ANO1, β-catenin, MMP9, snail, and E-cadherin were observed in the cytoplasmic membrane, cytoplasm, and nuclei of tumor cells and the expression of cyclin D1 was observed in the nuclei of tumor cells (Figure 1). Overall expression of immunohistochemical markers in tumor cells were evaluated for the scoring for ANO1, MMP9, snail, and E-cadherin expression. Based on the prognostic effect of the nuclear expressions of β-catenin and cyclin D1 in human malignant tumors [28], the expression of β-catenin and cyclin D1 were scored for their nuclear expression. Receiver operating characteristic curve analysis was performed to discriminate BCAs into negative and positive subgroups for each marker at the most predictive point to estimate survival of BCA patients. BCA cases with immunohistochemical staining scores equal or greater than five were included in the ANO1-positive group (area under the curve; 0.681, *P* < 0.001) (Figure 1A). The cut-off points for the immunohistochemical staining for β-catenin and cyclin D1 were six and five, respectively. The cut-off points for the MMP9, snail, and E-cadherin expression were seven (Figure 1). With these cut-off values, the expressions of ANO1, β-catenin, cyclin D1, MMP9, snail, and E-cadherin were grouped as positive in 51% (71 of 139), 38% (53 of 139), 41.7 (58/139), 58% (81 of 139), 42% (58 of 139), and 67% (93 of 139) of BCAs, respectively. Expression of ANO1 was significantly associated with distant metastatic relapse (*P* < 0.001), latent bone metastasis (*P* = 0.011), and β-catenin expression (*P* = 0.002), cyclin D1 expression (*P* = 0.004), MMP expression (*P* = 0.023), and snail expression (*P* = 0.001) (Table 1). ANO1 positivity was significantly associated with decreased expression of E-cadherin (*P* = 0.002). There was a possible correlation between ANO1 positivity and HER2 expression (*P* = 0.099). Expression of β-catenin was significantly associated with histologic type of BCA, mitotic count, histologic grade, cyclin D1 expression, and MMP9 expression. Expression of cyclin D1 was significantly associated with distant metastatic relapse, latent bone metastasis, ER expression, PR expression, and MMP9 expression. MMP9 expression was significantly associated with lymph node metastasis, histologic type, mitotic count, histologic grade, and snail expression. Snail expression was significantly associated with lymph node metastasis and distant metastatic relapse. E-cadherin negativity was significantly associated with distant metastatic relapse, latent bone metastasis, and histologic type (Table 1).

**Table 1:** Association between clinicopathological variables and immunohistochemical expression of ANO1, β-catenin, cyclin D1, MMP9, snail, and E-cadherin in breast carcinomas

Characteristics		No.	ANO1		β-catenin		Cyclin D1		MMP9		Snail		E-cadherin	
			Positive	*P*	Positive	*P*	Positive	*P*	Positive	*P*	Positive	*P*	Negative	*P*
Age, y	<50	98	51 (52%)	0.726	37 (38%)	0.888	41 (42%)	0.968	60 (61%)	0.275	40 (41%)	0.737	27 (28)	0.032
	≥50	41	20 (49%)		16 (39%)		17 (41%)		21 (51%)		18 (44%)		19 (46%)	
TNM stage	I	25	15 (60%)	0.251	10 (40%)	0.623	11 (44%)	0.965	11 (44%)	0.275	7 (28%)	0.229	6 (24%)	0.439
	II	95	44 (46%)		34 (36%)		39 (41%)		58 (61%)		41 (43%)		32 (34%)	
	III and IV	19	12 (63%)		9 (47%)		8 (42%)		12 (63%)		10 (53%)		8 (42%)	
T stage	1	37	24 (65%)	0.107	15 (41%)	0.910	16 (43%)	0.862	20 (54%)	0.201	15 (41%)	0.975	12 (32%)	0.995
	2	93	44 (47%)		35 (38%)		39 (42%)		58 (62%)		39 (42%)		31 (33%)	
	3 and 4	9	3 (33%)		3 (33%)		3 (33%)		3 (33%)		4 (44%)		3 (33%)	
Lymph node metastasis	Absence	77	38 (49%)	0.650	27 (35%)	0.407	31 (40%)	0.696	39 (51%)	0.042	23 (30%)	0.002	22 (29%)	0.207
	Presence	62	33 (53%)		26 (42%)		27 (44%)		42 (68%)		35 (56%)		24 (39%)	
Distant metastatic relapse	Absence	104	43 (41%)	<0.001	35 (34%)	0.061	36 (35%)	0.003	56 (54%)	0.068	38 (37%)	0.032	28 (27%)	0.008
	Presence	35	28 (80%)		18 (51%)		22 (63%)		25 (71%)		20 (57%)		18 (51%)	
Latent bone metastasis	Absence	129	62 (48%)	0.011	49 (38%)	0.899	50 (39%)	0.011	74 (57%)	0.435	53 (41%)	0.582	39 (30%)	0.010
	Presence	10	9 (90%)		4 (40%)		8 (80%)		4 (40%)		5 (50%)		7 (70%)	
Histologic type	NST	131	67 (51%)	0.950	53 (40%)	0.022	54 (41%)	0.625	79 (60%)	0.049	57 (44%)	0.084	38 (29%)	<0.001
	Lobular	8	4 (50%)		0 (0%)		4 (50%)		2 (25%)		1 (13%)		8 (100%)	
Mitoses/10 HPF	0–9	88	41 (47%)	0.375	25 (28%)	0.008	36 (41%)	0.096	41 (47%)	<0.001	34 (39%)	0.115	29 (33%)	0.611
	10–19	26	15 (58%)		15 (58%)		15 (58%)		19 (73%)		9 (35%)		7 (27%)	
	>19	25	15 (60%)		13 (52%)		7 (28%)		21 (84%)		15 (60%)		10 (40%)	
Histologic grade	1	48	23 (48%)	0.412	13 (27%)	0.022	20 (42%)	0.365	19 (40%)	<0.001	15 (31%)	0.11	14 (29%)	0.720
	2	62	30 (48%)		23 (37%)		29 (47%)		37 (60%)		27 (44%)		21 (34%)	
	3	29	18 (62%)		17 (59%)		9 (31%)		25 (86%)		16 (55%)		11 (38%)	
HER2	Negative	95	44 (46%)	0.099	33 (35%)	0.226	35 (37%)	0.086	54 (57%)	0.615	40 (42%)	0.894	30 (32%)	0.577
	Positive	44	27 (61%)		20 (45%)		25 (57%)		27 (61%)		18 (41%)		16 (36%)	
ER	Negative	62	32 (52%)	0.910	28 (45%)	0.126	16 (26%)	<0.001	38 (61%)	0.517	26 (42%)	0.964	20 (32%)	0.851
	Positive	77	39 (51%)		25 (32%)		42 (55%)		43 (56%)		32 (42%)		26 (34%)	
PR	Negative	58	32 (55%)	0.414	22 (38%)	0.967	14 (24%)	<0.001	32 (55%)	0.530	27 (47%)	0.329	22 (38%)	0.305
	Positive	81	39 (48%)		31 (38%)		44 (54%)		49 (60%)		31 (38%)		24 (30%)	
E-cadherin	Negative	46	32 (70%)	0.002	17 (37%)	0.841	21 (46%)	0.509	25 (54%)	0.509	16 (35%)	0.243		
	Positive	93	39 (42%)		36 (39%)		37 (40%)		56 (60%)		42 (45%)			
Snail	Negative	81	32 (40%)	0.001	26 (32%)	0.084	34 (42%)	0.944	35 (43%)	<0.001				
	Positive	58	39 (67%)		27 (47%)		24 (41%)		46 (79%)					
MMP9	Negative	58	23 (40%)	0.023	10 (17%)	<0.001	15 (26%)	0.001						
	Positive	81	48 (59%)		43 (53%)		43 (53%)							
Cyclin D1	Negative	81	33 (41%)	0.004	24 (30%)	0.015								
	Positive	58	38 (66%)		29 (50%)									
β-catenin	Negative	86	35 (41%)	0.002										
	Positive	53	36 (68%)											

NST, invasive carcinoma of no special type; HPF, high-power fields; ER, estrogen receptor; PR, progesterone receptor.

**Figure 2:**
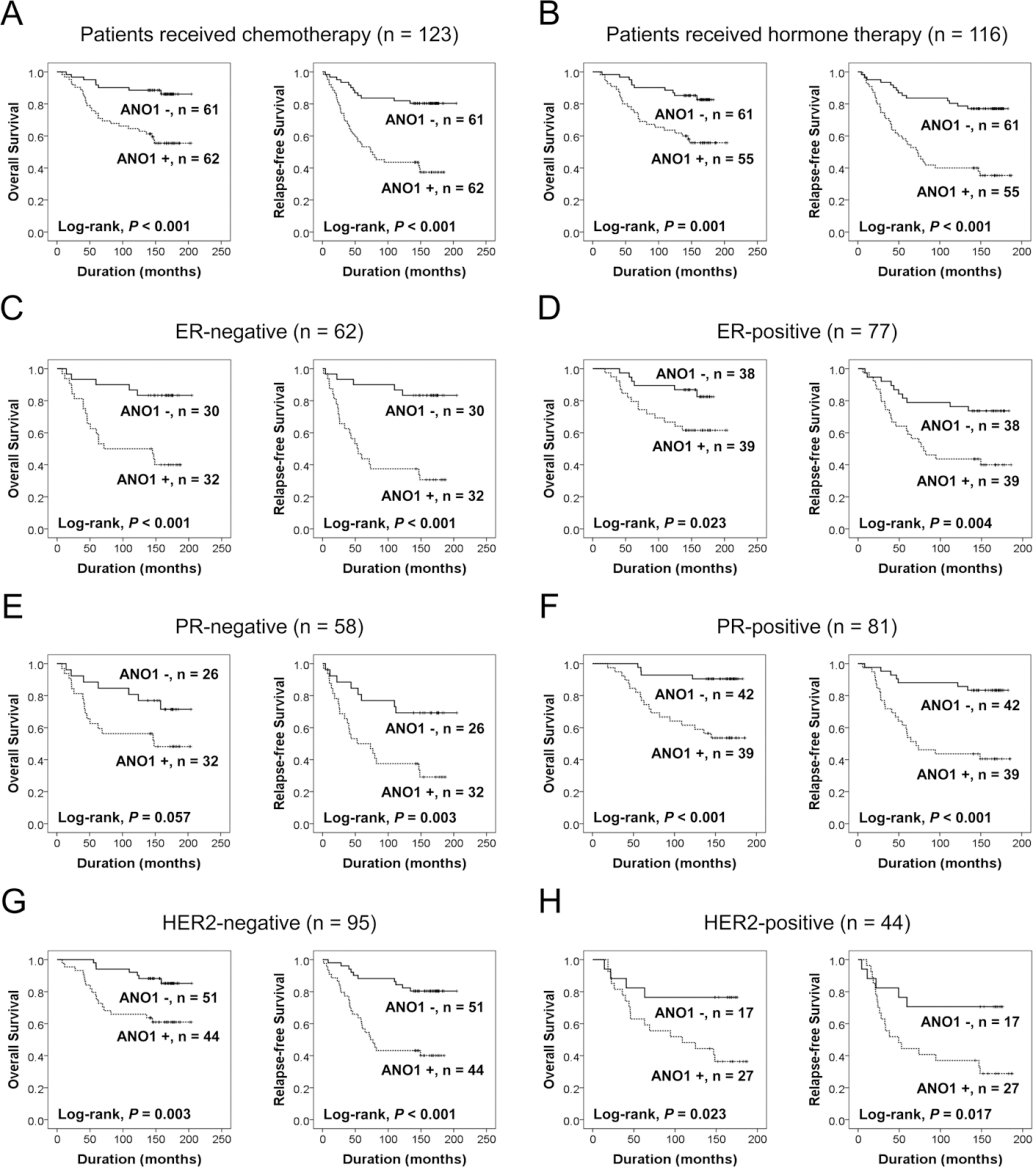
Survival analysis for the expression of ANO1 in subgroups of breast carcinoma patients (**A–H**) Kaplan-Meier survival analysis for the overall survival and relapse-free survival according to the ANO1 expression in subgroups of breast carcinoma patients who received adjuvant chemotherapy (A) or endocrine therapy (B) and estrogen receptor-negative (C), estrogen receptor-positive (D), progesterone receptor-negative (E), progesterone receptor-positive (F), HER2-negative (G), and HER2-positive (H) subgroups.

When univariate Cox proportional regression analysis was performed for the survival of BCA patients, older age of patients (*P* < 0.001), higher TNM stage (*P* = 0.045), higher histologic grade (*P* = 0.024), HER2 expression (*P* = 0.006), ANO1 positivity (*P* < 0.001), β-catenin positivity (*P* = 0.014), cyclin D1 positivity (*P* = 0.043), snail positivity (*P* = 0.002), MMP9 positivity (*P* < 0.001), and loss of expression of E-cadherin (*P* < 0.001) were significantly associated with shorter overall survival (OS) (Table 2). The factors significantly associated with shorter relapse-free survival (RFS) of BCA patients were older age of patients (*P* = 0.014), HER2 expression (*P* = 0.036), ANO1 positivity (*P* < 0.001), cyclin D1 positivity (*P* = 0.026), MMP9 positivity (*P* = 0.012), and E-cadherin negativity (*P* < 0.001) (Table 2). The survival curves for OS and RFS of each marker are presented in Figure 1. ANO1 positivity was significantly associated with overall survival (Log-rank, *P* < 0.001) and relapse-free survival (Log-rank, *P* < 0.001) (Figure 1). In addition, we further analyzed the prognostic significance of ANO1 expression in various subgroups of BCA patients according to type of adjuvant therapy and the expression status of ER, PR, and HER2 (Table 3). ANO1 positivity predicted a 4.032-fold [95% confidence interval (95% CI); 1.830–8.886, *P* < 0.001] greater risk of death and a 4.272-fold (95% CI; 2.227–8.194, *P* < 0.001) greater risk of relapse or death in the patients who received adjuvant chemotherapy. Among the subgroups of BCA patients who received adjuvant hormone therapy, ANO1 positivity was significantly associated with shorter OS [hazard ratio (HR); 3.227, 95% CI; 1.542–6.754, *P* = 0.002] and RFS (HR; 3.871, 95% CI; 2.076–7.218, *P* < 0.001). In addition, ANO1 positivity was significantly associated with shorter survival in ER-negative (OS; *P* = 0.002, RFS; *P* < 0.001), ER-positive (OS; *P* = 0.030, RFS; *P* = 0.006), PR-positive (OS; *P* = 0.001, RFS; *P* < 0.001), HER2-negative (OS; *P* = 0.006, RFS; *P* < 0.001), and HER2-positive (OS; *P* = 0.032, RFS; *P* = 0.023) subgroups of BCA patients (Figure 2) (Table 3).

**Table 2:** Univariate cox regression analysis for the overall survival and relapse-free survival of 139 breast carcinomas

Characteristics	No.	OS		RFS	
		HR (95% CI)	*P*	HR (95% CI)	*P*
Age, y, ≥50 (*vs*. <50)	41/139	2.903 (1.616–5.216)	<0.001	1.907 (1.140–3.190)	0.014
TNM stage, I	25/139	1	0.045	1	0.429
II	95/139	1.718 (0.667–4.429)	0.262	1.321 (0.642–2.718)	0.450
III and IV	19/139	3.532 (1.205–10.346)	0.021	1.813 (0.736–4.463)	0.196
Histologic grade, 1	48/139	1	0.024	1	0.324
2	62/139	1.387 (0.660–2.915)	0.388	1.060 (0.584–1.926)	0.847
3	29/139	2.801 (1.286–6.104)	0.010	1.597 (0.821–3.106)	0.168
HER2, positive (*vs*. negative)	44/139	2.267 (1.261–4.074)	0.006	1.741 (1.038–2.921)	0.036
ER, positive (*vs*. negative)	77/139	0.637 (0.354–1.144)	0.131	0.952 (0.572–1.584)	0.850
PR, positive (*vs*. negative)	81/139	0.601 (0.335–1.079)	0.088	0.627 (0.378–1.041)	0.071
ANO1, positive (*vs*. negative)	71/139	3.718 (1.881–7.346)	<0.001	4.005 (2.226–7.203)	<0.001
β-catenin, positive (*vs.* negative)	53/139	2.090 (1.163–3.755)	0.014	1.595 (0.960–2.650)	0.071
Cyclin D1, positive (*vs.* negative)	58/139	1.836 (1.019–3.307)	0.043	1.778 (1.070–2.956)	0.026
MMP9, positive (*vs.* negative)	81/139	3.615 (1.740–7.513)	<0.001	2.024 (1.164–3.517)	0.012
Snail, positive (*vs.* negative)	58/139	2.622 (1.443–4.764)	0.002	1.572 (0.947–2.610)	0.080
E-cadherin, negative (*vs.* positive)	46/139	2.883 (1.600–5.195)	<0.001	2.992 (1.794–4.990)	<0.001

OS, overall survival; RFS, relapse-free survival; ER, estrogen receptor; PR, progesterone receptor.

**Table 3:** Univariate cox regression analysis for the prognostic significance of ANO1 expression in the subgroups of the breast carcinoma patients according to type of adjuvant therapy, and the expression of ER, PR, and HER2

Subgroups of breast carcinomas		No.	OS, ANO1 positive (*vs.* negative)		RFS, ANO1 positive (*vs.* negative)	
			HR (95% CI)	*P*	HR (95% CI)	*P*
Adjuvant chemotherapy		123	4.032 (1.830–8.886)	<0.001	4.272 (2.227–8.194)	<0.001
Adjuvant hormone therapy		116	3.227 (1.542–6.754)	0.002	3.871 (2.076–7.218)	<0.001
ER	Negative	62	4.787 (1.782–12.865)	0.002	6.257 (2.355–16.627)	<0.001
	Positive	77	2.860 (1.108–7.377)	0.030	2.866 (1.361–6.035)	0.006
PR	Negative	58	2.320 (0.952–5.653)	0.064	3.172 (1.405–7.162)	0.005
	Positive	81	6.040 (2.041–17.867)	0.001	4.826 (2.064–11.283)	<0.001
HER2	Negative	95	3.467 (1.436–8.368)	0.006	4.213 (2.025–8.766)	<0.001
	Positive	44	3.301 (1.106–9.853)	0.032	3.148 (1.168–8.482)	0.023

OS, overall survival; RFS, relapse-free survival; ER, estrogen receptor; PR, progesterone receptor.

Multivariate analysis was performed with the factors significantly associated with OS or RFS: age of patients, TNM stage, histologic grade, and the expression of HER2, ANO1, β-catenin, cyclin D1, MMP9, snail, and E-cadherin. Multivariate analysis revealed that age of patients (OS; *P* < 0.001, RFS; *P* = 0.045) and ANO1 positivity (OS; *P* < 0.001, RFS; *P* < 0.001) were independent predictors for shorter OS and RFS of BCA patients. The patients with BCA which were ANO1-positive had a 3.217-fold (95% CI; 1.609–6.434) greater risk of death and a 3.411-fold (95% CI; 1.854–6.277) greater risk of relapse or death compared with patients who had ANO1-negative BCA. MMP expression was an independent indicator of poor prognosis for OS (*P* = 0.002) and loss of E-cadherin expression was significantly associated shorter RFS (*P* = 0.018) (Table 4).

**Table 4:** Multivariate cox regression survival analysis for overall survival and relapse–free survival of 139 breast carcinomas

Characteristics	OS		RFS	
	HR (95% CI)	*P*	HR (95% CI)	*P*
Age, y, ≥50 (*vs*. <50)	3.370 (1.868–6.080)	<0.001	1.723 (1.013–2.932)	0.045
ANO1, positive (*vs.* negative)	3.217 (1.609–6.434)	<0.001	3.411 (1.854–6.277)	<0.001
MMP9, positive (*vs.* negative)	3.298 (1.565–6.947)	0.002		
E–cadherin, negative (*vs.* positive)			1.927 (1.118–3.322)	0.018

OS, overall survival; RFS, relapse–free survival; ER, estrogen receptor; PR, progesterone receptor. The factors included in multivariate analysis were age of patients, TNM stage, histologic grade, and expression of HER2, ANO1, β–catenin, cyclin D1, snail, MMP9, and E–cadherin.

### Inhibition of ANO1 suppresses the growth and invasiveness of BCA cells

**Figure 3:**
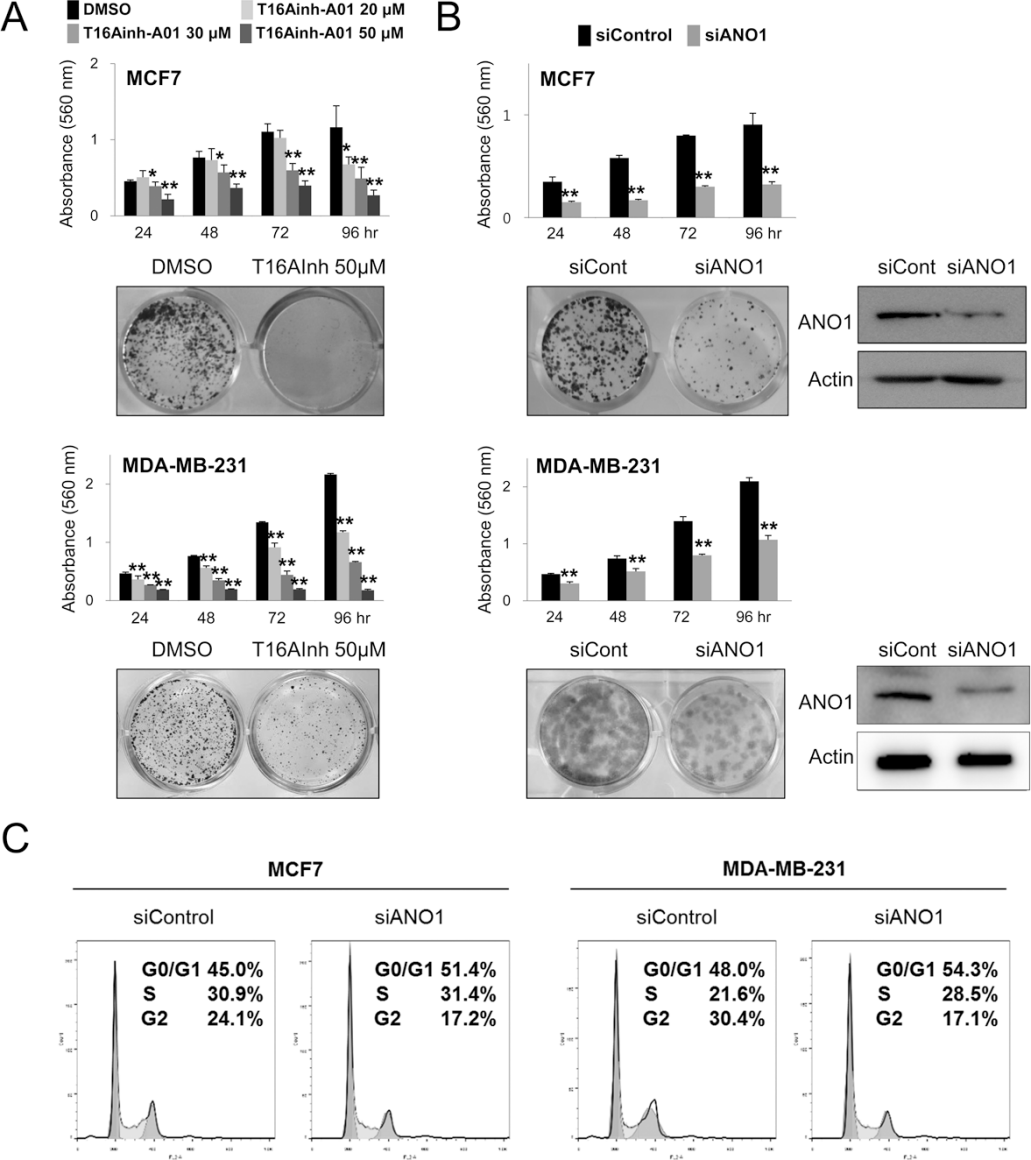
Inhibition of ANO1 decreases the proliferation of breast cancer cells (**A**) Treatment of T16Ainh-A01, an ANO1 inhibitor, significantly inhibited the proliferation of both MCF7 and MDA-MB-231 cells in a dose-dependent manner. (**B**) MTT and colony-forming assays demonstrate that the knock-down of ANO1 with siRNA for ANO1 inhibited the proliferation of both MCF7 and MDA-MB-231 cells. (**C**) Flow-cytometry cell cycle analysis demonstrates that the G0/G1 population increases with knock-down of ANO1. ^*^P < 0.05; ^**^P < 0.001.

**Figure 4:**
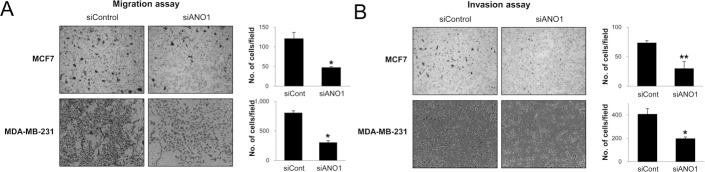
The knock-down of ANO1 decreases the invasiveness of breast cancer cells (**A** and **B**) The migration (A) and invasion (B) of MCF7 and MDA-MB-231 cells were significantly decreased with knock-down of ANO1. ^*^P < 0.05; ^**^P < 0.001.

Because the expression of ANO1 in human BCA tissue samples was associated with latent metastasis and shorter survival of diagnosed BCA patients, we evaluated the effect of ANO1 expression on the growth and invasiveness of BCA cells. The inhibitor of ANO1, T16Ainh-A01, inhibited proliferation of MCF7 and MDA-MB-231 BCA cells is a dose-dependent manner (Figure 3A). In addition, knock-down of ANO1 with siRNA for ANO1 also inhibited the proliferation of both MCF7 and MDA-MB-231 BCA cells (Figure 3B). The effects of the knock-down of ANO1 on the proliferation of BCA cells were related with G0/G1 arrest (Figure 3C). In addition, migration and invasion chamber assays demonstrated that the invasive activity of BCA cells was significantly inhibited by a knock-down of ANO1 in both MCF7 and MDA-MB-231 BCA cells (Figure 4).

### Inhibition of ANO1 suppresses the signaling molecules associated with the proliferation and EMT of BCA cells

**Figure 5:**
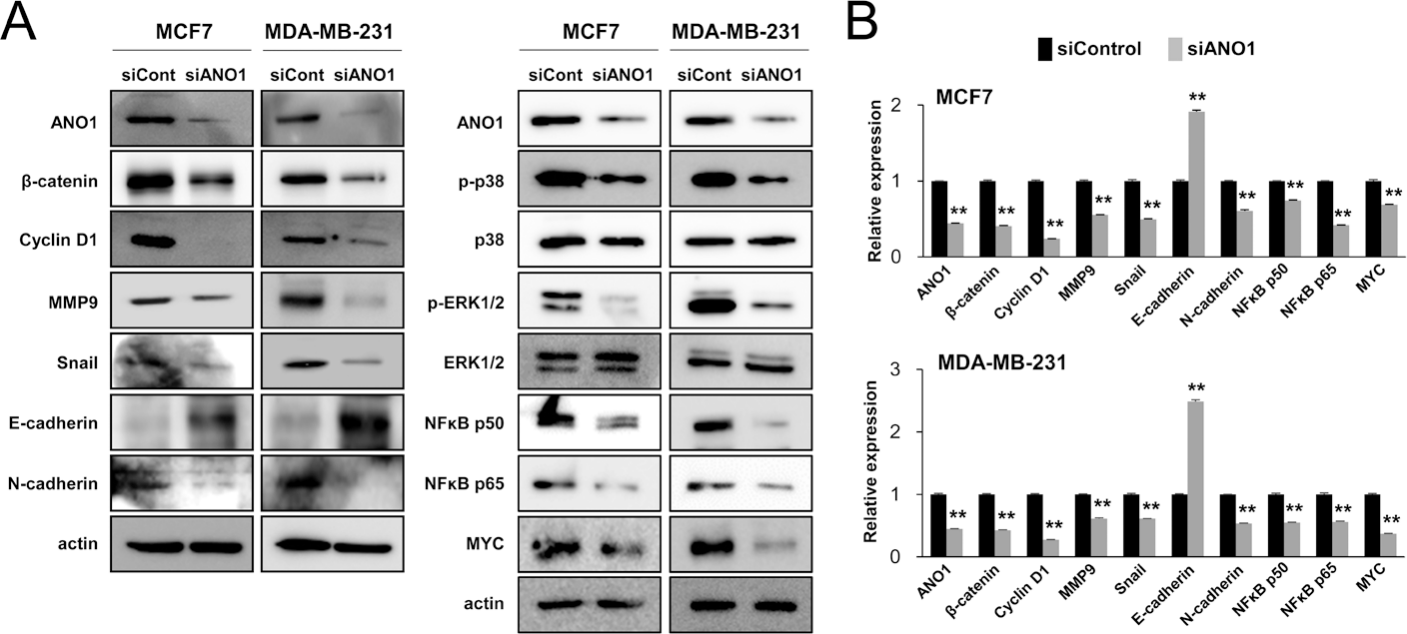
The expression of ANO1 is associated with the expression of signaling molecules associated with the proliferation and invasiveness of breast cancer cells (**A**) The protein levels of β-catenin, cyclin D1, MMP9, snail, N-cadherin, phospho-p38 MAPK, phospho-ERK1/2, NFκB p50, NFκB p65, and MYC decreased with knock-down of ANO1 by siRNA for ANO1 in both MCF7 and MDA-MB-231 cells. In contrast, the expression of the protein of E-cadherin increased with knock-down of ANO1 in both MCF7 and MDA-MB-231 cells. (**B**) The mRNA levels of β-catenin, cyclin D1, MMP9, snail, N-cadherin, NFκB p50, NFκB p65, and MYC decreased, but the expression of E-cadherin mRNA increased with knock-down of ANO1 in both MCF7 and MDA-MB-231 cells. ^**^P < 0.001.

As demonstrated in Figures 3 and 4, ANO1 expression was significantly associated with both the proliferation and invasiveness of BCA cells. Therefore, we examined the expression of signaling molecules related to proliferation or invasiveness of cancer cells. The knock-down of ANO1 suppressed expression of mRNA and protein of β-catenin and cyclin D1, which are involved in cell cycle progression from G0/G1 phase (Figure 5). In addition, knock-down of ANO1 inhibited expression of mRNA and protein of MMP9, snail, N-cadherin, NFκB p50, NFκB p65, and MYC, but increased expression of E-cadherin in both MCF7 and MDA-MB-231 BCA cells. In addition, phosphorylation of p38 MAPK and ERK1/2 were decreased with knock-down of ANO1 (Figure 5).

## DISCUSSION

In this study, we demonstrate that the expression of ANO1 is closely associated with latent distant metastasis of BCAs and that ANO1 positivity is an independent indicator of poor prognosis for the OS and RFS of BCA patients. In line with our results, higher expression of ANO1 was associated with shorter OS of gastric carcinoma [9], head and neck squamous cell carcinoma [14], and esophageal squamous cell carcinoma patients [29]. Although it is not statistically significant, immunohistochemical expression of ANO1 suggested a tendency towards shorter disease-free survival of BCA patients [21]. In contrast, ANO1 expression was associated with longer survival of PR-positive or HER2-negative subgroups of BCAs [22, 27]. In addition, the prognostic significance of immunohistochemical expression of ANO1 differed between HPV-positive and HPV-negative head and neck squamous cell carcinomas. ANO1 expression was significantly associated with shorter survival of patients in only the HPV-negative cancers (Log-rank, *P* = 0.005) but not in HPV-positive cancers (Log-rank, *P* = 0.560) [30]. These findings suggest that biologic and prognostic effects of ANO1 might be different according to the type of cells and/or underlying mechanism of tumorigenesis. However, when we evaluated prognostic significance of ANO1 expression in various subgroups of BCAs according to the expression of ER, PR, or HER2, the expression of ANO1 was significantly associated with shorter OS and RFS in PR-positive, PR-negative, HER2-positive, HER2-negative, ER-positive, or ER-negative BCA subgroups (Table 3). Therefore, despite conflicting reports in the literature, our results suggest that ANO1 expression might be a potential prognostic marker of BCA patients and could be a potential therapeutic target. However, further study is needed to clarify the roles and clinical significance of the expression of ANO1 in human malignant tumors.

In addition to the role of ANO1 in the survival of BCA patients, ANO1 expression was associated with the proliferation of BCA cells. Consistently, ANO1-mediated proliferation of cancer cells has been reported in various types of cancer cells. Regarding the mechanism how ANO1 regulates cellular proliferation, it has been suggested that ANO1 is involved in the proliferation of cells by regulating intracellular concentrations of chloride *via* its role as a calcium-activated chloride channel. Chloride channel blockers inhibited proliferation of ANO1-expressing pancreatic cancer cells [31]. In addition, ANO1 could act as a signaling molecule regulating various intracellular components. The signaling pathways involved in the proliferation of cells, such as MAPK-ERK1/2, cyclin D1, EGFR, NFκB, and MYC, have been influenced by the ANO1 [9, 12, 17, 18, 25]. ANO1 is also involved in the regulation of cell cycle- and apoptosis-regulating molecules [10, 12, 17, 25, 32]. Our results also demonstrated that ANO1 is involved in the proliferation of BCA cells by regulating the expression of β-catenin and cyclin D1 and knock-down of ANO1 caused G0/G1 cell cycle arrest. In BCA cells, knock-down of ANO1 decreased expression of MMP9, snail, N-cadherin, phospho-p38 MAPK, phospho-ERK1/2, NFκB p50, NFκB p65, and MYC. In BCA tissue samples, ANO1 positivity was significantly associated with positive expression of β-catenin, cyclin D1, MMP9, and snail (Table 1). In addition, the role of ANO1 as a signaling molecule could is also suggested by its localization within the cells. The expression of ANO1 was not restricted to the cytoplasmic membrane, but was expressed in both the cytoplasm and nuclei of tumor cells [11, 13, 22, 27, 30].

One interesting finding of this study is that ANO1 positivity was an independent indicator of poor prognosis of BCA patients. However, there was no close association between ANO1 expression and variable clinicopathological factors related with the prognosis of BCA patients, such as TNM stage, lymph node metastasis, histologic grade, and the expression of ER, PR, and HER2. Moreover, although it was not statistically significant, ANO1 positivity was low in high T stage compared with low T stage (ANO1 positive rate: T stage 1; 65%, T stage 2; 47%, T stage 4; 33%). These findings are discordant to our *in vitro* results that ANO1 is associated with cellular proliferation. Similarly, there are inconsistent reports regarding the role of ANO1 in the proliferation of cancer cells [9, 10, 14, 15, 17, 18, 20]. Despite involvement of ANO1 in cell motility, inhibition of ANO1 did not influence the proliferation of cancer cells [9, 14, 20]. These findings suggest that the role of ANO1 in cellular proliferation is not sufficient to explain the prognostic role of ANO1 expression in cancer patients. Therefore, other roles of ANO1 other than for the proliferation of cells might be responsible for the progression of ANO1-expressing cancers.

In our study, interestingly, ANO1 positivity was significantly associated with relapse of BCAs, higher expression of MMP9 and snail, and loss of E-cadherin expression. Moreover, higher expression levels of ANO1, MMP9, and snail and loss of E-cadherin expression were significantly associated with shorter survival of BCA patients. In addition, the expression of MMP9, snail, and N-cadherin were decreased with knock-down of ANO1 and the expression of E-cadherin increased. In line with our results, decreased migration and invasion activity with inhibition of ANO1 have been reported in gastric carcinoma [9], hepatocellular carcinoma [10], lung cancer [15], oral squamous cell carcinoma [13], prostatic carcinoma [32], colorectal carcinoma [17], and glioma [12]. Acquiring an invasive phenotype is closely related with EMT and the involvement of ANO1 in EMT has been suggested by its modulation of the secretion of TGF-β and resultant repression of E-cadherin expression in gastric cancer cells [9]. ANO1 is also involved in cell migration and invasion by regulating the expression of MMP2 and MMP9 in glioma cells [12]. Our result also demonstrate that ANO1 inhibition significantly inhibits invasiveness of BCA cells and suppresses an EMT-phenotype in BCA cells. Collectively, these results suggest that ANO1 might be an inducer of EMT that is associated with invasiveness of cancer cells and/or involved in resistance to the initial therapies. In addition, when considering that the morbidity and mortality in BCA patients are mainly due to invasion and metastasis of the primary tumor [33], controlling of ANO1 might be a potential target for the treatment of BCA patients. However, there are controversial reports on the role of ANO1 in the invasiveness of cells. In head and neck squamous cell carcinomas, inhibition of ANO1 increased migration activity, which was associated with decreased expression of E-cadherin [19]. Moreover, the EMT-phenotype of metastatic lesion in lymph node was associated with decreased expression of ANO1 [19]. In this context, the function of ANO1 was influenced by methylation of the ANO1 promoter, resulting decreased expression of ANO1 [19].

Poor prognosis of ANO1-expressing cancers might be explained by the fact that EMT is closely associated with drug resistance and/or stemness of cancer cells [34]. In our results, ANO1 expression was significantly associated with latent metastasis (distant metastatic relapse; *P* < 0.001, latent bone metastasis; *P* = 0.011) of BCA, suggesting that ANO1 might be involved in the progression of cancer after initial treatment. Moreover, ANO1 expression was also significantly associated with shorter OS and RFS in the subgroups of BCA patients who received adjuvant endocrine therapy or chemotherapy. Therefore, these findings suggest that ANO1 might be involved in the acquisition of resistance to these types of therapy. ANO1-mediated resistance to therapy might be related with EMT-related molecules, such as E-cadherin and snail. Because the expression of E-cadherin controls the sensitivity of EGFR kinase inhibitors [35], re-expression of E-cadherin by ANO1 inhibition, as shown by this report and others, could be an effective strategy to overcome drug resistance. Snail is also suggested as one of the main regulators of chemoresistance and stemness in BCA cells [36], and our results show that inhibition of ANO1 decreases expression of snail. In addition, although there is no definitive report regarding the role of ANO1 in the stemness of cancer cells, it is suspected based upon the result that ANO1 induced expression of MYC [12]. The potential of therapeutic efficacy of ANO1 inhibition on drug resistance has been suggested in trastuzumab-resistant cells. Pharmacological and siRNA-mediated inhibition of ANO1 suppressed HER2 transcription in HER2-positive YMB-1 BCA cells have acquired resistance to HER2 inhibitor trastuzumab [26].

As a therapeutic target of human malignant tumors, inhibition of ANO1 is under evaluation in various cancers. Despite limited data on the mechanism how ANO1 is involved in tumorigenesis, many previous reports demonstrate that inhibition of ANO1 is sufficient to inhibit tumor growth *in vitro* and *in vivo* [10, 12, 15–18, 25, 32]. The shRNA- or siRNA-mediated inhibition of ANO1 suppressed proliferation and invasiveness of human BCA cells [18], lung cancer cells [15], colorectal cancers [17], prostatic cancers [11, 32], hepatocellular carcinomas [10], and head and neck squamous cell carcinomas [25]. Chemical compounds inhibiting ANO1, such as CaCCinh-A01 and T16Ainh-A01 also inhibited proliferation and invasion of human cancer cells [16–18, 25, 32, 37]. In addition, although there are limited reports, the control of cancer metastasis could be archived by the regulation of ANO1. Treatment of microRNA-381 inhibits metastasis of gastric carcinomas by suppressing ANO1 [38]. In this study, the inhibition of ANO1 with T16Ainh-A01 or siRNA for ANO1 inhibited proliferation of both MCF7 and MDA-MB-231 BCA cells. In addition, siRNA-mediated inhibition of ANO1 induced G0/G1 arrest and inhibited the migration and invasion activity of BCA cells. Therefore, inhibition of ANO1 might be a therapeutic strategy for the treatment of human malignant tumors. However, the effect of ANO1 on the cellular proliferation differed according to the phenotype of cancer cells. ANO1 promoted proliferation of ER^+^/PR^+^/HER2^-^ MCF7 cells but inhibited proliferation of ER^-^/PR^-^/HER2^-^ MDA-MB-435S cells [27]. Therefore, further study is needed to determine if ANO1 could be a new therapeutic target in treatment of human malignant tumors.

In conclusion, this study demonstrates that ANO1 expression is an independent indicator of shorter OS and RFS of BCA patients. In addition, pharmacologic and siRNA-mediated inhibition of ANO1 suppressed cellular proliferation and invasiveness, which were associated with suppression of cellular proliferation and EMT-related signaling molecules, such as β-catenin, cyclin D1, snail, N-cadherin, and MMP9. Therefore, this study suggests that ANO1 expression could be used as an indicator of poor prognosis of BCA patients and ANO1 might be a therapeutic target for the poor prognostic subgroup of BCA patients who have ANO1-positive tumors.

## MATERIALS AND METHODS

### Breast carcinoma patients and tissue samples

This study received approval from the Institutional Reviewer Board of Chonbuk National University Hospital (IRB No.; CUH 2016-09-012) and the requirement for informed consent was waived due to the retrospective nature of this study. All experiments were performed in accordance with relevant guidelines and regulations.

One hundred and thirty-nine cases of invasive BCAs who underwent therapeutic operation between January 1997 and December 2002 were used in this study. Clinical information was obtained by reviewing medical records and pathologic reports. The median age of the patients was 44 years (range; 22–72 years). 123 patients received adjuvant chemotherapy, 83 patients received postoperative radiation therapy, and 116 patients received hormone therapy. The median follow-up duration was 154.1 months (range; 7.7–204.6 months). The cases with complete medical records, original histologic slides, and tissue blocks were included in this study, and histologic slides were reviewed. The BCAs were classified according to the age of patients, TNM stage based on American Joint Committee on Cancer staging system [39], lymph node metastasis, distant metastatic relapse, latent bone metastasis, histologic type, and histologic grade, were assessed according to the World Health Organization classification [40], mitotic counts, and the expression of HER2, estrogen receptor (ER), and progesterone receptor. Tissue microarray recipient blocks with 3.0 mm cores were used, and one core was obtained per case from the most representative area composed of intact BCA cells.

### Immunohistochemical staining and evaluation

The tissue sections were de-paraffinized and boiled with antigen retrieval solution (pH 6.0, DAKO, Glostrup, Denmark) for 20 minutes in a microwave oven. Primary antibody for ANO1 (1:100. Abcam, Cambridge, MA), β-catenin (1:100. BD Biosciences, San Jose, CA), cyclin D1 (1:50. Thermo Fisher Scientific, Fremont, CA), MMP9 (1:50. Santa Cruz Biotechnology, Santa Cruz, CA), snail (1:100. Abcam, Cambridge, MA), and E-cadherin (1:100. BD Biosciences, San Jose, CA) were used for the immunohistochemical staining. Stained slides were evaluated by two pathologists (KYJ and SJN) without information of the patients. Immunohistochemical staining slides were scored by the sum of staining intensity (0; no staining, 1; weak staining, 2; moderate staining, 3; strong staining) and percentage of stained cells (0; 0%, 1; 1%, 2; 2–10%, 3; 11–33%, 4; 34–66%, 5; 67–100%) [41–44]. The immunohistochemical staining score ranged from zero to eight.

### Cell culture and chemicals

The human BCA cell lines, MCF7 and MDA-MB-231, were purchased from the Korean Cell Line Bank (KCLB, Seoul, Korea). The cell lines were maintained in RPMI 1640 and DMEM medium supplemented with 10% fetal bovine serum (FBS) and penicillin/streptomycin (100 U/ml) (Gibco BRL, Gaithersburg, MD) at 37°C in a humidified 5% CO2 incubator. As an ANO1 inhibitor, T16Ainh-A01 (Sigma, St. Louis, MO) was used.

### Transfection of siRNA (RNA interference)

The siRNAs for negative control and ANO1 were purchased from Santa Cruz Biotechnology (Santa Cruz Biotechnology, Santa Cruz, CA). Lipofectamine RNAiMAX or Lipofectamine 2000 (Invitrogen, Carlsbad, CA) was used for the transfection according to the manufacturer’s instructions.

### Cell proliferation assay

The cell proliferation studies were measured using a 3-(4,5-dimethylthiazol -2-yl)-2,5-diphenyltetrazonium bromide (MTT) (Sigma, St. Louis, MO) assay and a colony-forming assay. For the MTT assay, each 5 × 10^3^ MCF7 cells and 3 × 10^3^ MDA-MB-231 cells with siRNAs or inhibitor were seeded in 96-well culture plates for 24, 48, 72, or 96 hours, and the absorbance was measured using a microtiter plate reader (Bio-Rad, Richmond, CA) at 560 nm. For the colony-forming assay, 5 × 10^3^ MCF7 and 3 × 10^3^ MDA-MB-231 cells were cultured in 12-well culture plates for 10 days. After 10 days, the colonies were fixed with methanol and stained with methylene blue.

### Western blotting

To get protein lysate from the cells, the cells were lysed with PRO-PREP Protein Extraction Solution (iNtRON Biotechnology Inc., Korea) containing 1x phosphatase inhibitor cocktails 2, 3 (Sigma, St. Louis, MO). Proteins were separated on 10% SDS-PAGE and were then transferred to a PVDF membrane. The membranes were incubated with primary antibodies for ANO1 (Abcam, Cambridge, MA), β-catenin (BD Biosciences, San Jose, CA), cyclin D1 (Cell Signaling Technology, Beverly, MA), MMP9 (Thermo Fisher Scientific, Fremont, CA), snail (Abcam, Cambridge, UK), E-cadherin (BD Biosciences, San Jose, CA), N-cadherin (BD Biosciences, San Jose, CA), p38 MAPK (Cell Signaling Technology, Beverly, MA), phospho-p38 MAPK (Cell Signaling Technology, Beverly, MA), ERK1/2 (Cell Signaling Technology, Beverly, MA), phospho-ERK1/2 (Cell Signaling Technology, Beverly, MA), NFκB p50 (Santa Cruz Biotechnology, Santa Cruz, CA), NFκB p50 (Santa Cruz Biotechnology, Santa Cruz, CA), NFκB p65 (Abcam, Cambridge, UK), MYC (Abcam, Cambridge, UK), or actin (Sigma, St. Louis, MO). The proteins were detected by a LAS-3000 luminescent image analyzer (Fuji Film, Tokyo, Japan).

### Cell cycle analysis

After transfection, cells were harvested, washed with 1X PBS and fixed in 70% ethanol at 4°C overnight. After washing twice with1X PBS, the cells were incubated with 1X PBS containing 50 μg/mL propidium iodide (Sigma, St. Louis, MO) and 50 μg/mL RNase A (Sigma, St. Louis, MO) at 37°C for 30 min. 10,000 cells were measured by a FACStar flow cytometer (Becton-Dickinson, San Jose, CA) and analyzed using Lysis ΙΙ and CellFIT software (Becton-Dickinson) or ModFit software (Verity Software House Inc., Topsham, ME).

### *In vitro* migration and invasion assays

*In vitro* migration and invasion abilities were measured in a 24-transwell chamber (Corning Life Sciences, Acton, MA) and a Matrigel-coated Invasion chamber (BD Biosciences, San Jose, CA). For the migration assay, each 1x10^5^ MCF7 and 5 × 10^4^ MDA-MB-231 cells with siRNA for ANO1 or negative controls were seeded in serum-free RPMI 1640 or DMEM medium into the upper chambers, and medium with 20% FBS was added to the bottom chamber as a chemoattractant. After 24 hours, the cells on the bottom side of the membrane were fixed and stained using a Diff-Quick solution. For the invasion assay, 3 × 10^5^ transfected-MCF7 and 1 × 10^5^ MDA-MB-231 cells in the medium with 2% FBS were seeded in the upper chamber of transwell inserts. Medium containing 20% serum as a chemoattractant was added to each well. After 48 hours, the cells that invaded into the bottom membrane were fixed and stained with a Diff-Quick solution. The migrating or invading cells to the lower surface of the filter were counted in five microscopic fields (magnification ×100) per well.

### Quantitative reverse-transcription polymerase chain reaction

RNA was isolated with the RNeasy Mini Kit (Qiagen Sciences, Valencia, CA) and reverse transcription of 1.5 μg RNA was performed with Taqman Reverse Transcription Reagents (Applied Biosystems, Foster City, CA). Quantitative reverse-transcription polymerase chain reaction was carried out using the Applied Biosystems Prism 7900HT Sequence Detection System and Sybr Green polymerase chain reaction Master Mix (Applied Biosystems, Foster City, CA). All experiments were performed in triplicate, and the results were normalized to the expression of glyceraldehyde-3-phosphate dehydrogenase (GAPDH), a reference housekeeping gene. Primer sequences for quantitative reverse-transcription polymerase chain reaction are listed in Table 5.

**Table 5:** Primer sequences used for quantitative real-time polymerase chain reactions

Gene	Primer Sequence Forward/Reverse	Product size	Accession number
*ANO1*	F: 5′-ATTTCACCAATCTTGTCTCCATCA-3′	368	NM_018043.5
	R: 5′-TGATAACTCCAAGAACGATTGCA-3′		
*SNAL1* (Snail)	F: 5′-GCACATCCGAAGCCACAC-3′	225	NM_005985.3
	R: 5′-GGAGAAGGTCGAGCACAC-3′		
*E-cadherin*	F: 5′-CCCGGGACAACGTTTATTAC-3′	72	NM_004360.3
	R: 5′-ACTTCCCCTTCCTCAGTGAT-3′		
*N-cadherin*	F: 5′-ACAGTGGCCACCTACAAAGG-3′	201	NM_001792.4
	R: 5′-CCGAGATGGGGTTGATAATG-3′		
*MMP9*	F: 5′-GACGCAGACATCGTCATCCA-3′	200	NM_004994.2
	R: 5′-GCCGCGCCATCTGCGTTTCCAAA-3′		
*CTNNB1* (β-catenin)	F: 5′-AAAATGGCAGTGCGTTTAG-3′	100	NM_001904.3
	R: 5′-TTTGAAGGCAGTCTGTCGTA-3′		
*CCND1* (Cyclin D1)	F: 5′-GCTGCGAAGTGGAAACCATC-3′	135	NM_053056.2
	R: 5′-CCTCCTTCTGCACACATTTGAA-3′		
*NFKB1* (NFκB p50)	F: 5′-ACAAATGGGCTACACCGAAG-3′	238	NM_003998.3
	R: 5′-ATGGGGCATTTTGTTGAGAG-3′		
*RELA* (NF*κ*B p65)	F: 5′-CTGAACCAGGGCATACCTGT-3′	197	NM_021975.3
	R: 5′-GAGAAGTCCATGTCCGCAAT-3′		
*MYC*	F: 5′-TTCGGGTAGTGGAAAACCAG-3′	203	NM_002467.4
	R: 5′-CAGCAGCTCGAATTTCTTCC-3′		
*GAPDH*	F: 5′-AACAGCGACACCCACTCCTC-3′	258	NM_001256799.1
	R: 5′-GGAGGGGAGATTCAGTGTGGT-3′		

Web link to accession numbers: https://www.ncbi.nlm.nih.gov/gene.

### Statistical analysis

To determine the cut-off point for ANO1 positivity immunohistochemical staining, Receiver operating characteristic curve analysis were performed [41, 43]. Survival analysis was performed for the OS and RFS. The follow-up end point was December 2013. The death of patients was an event of OS analysis and the patients who were alive at last follow-up date were considered censored. The relapse of BCA or death of patients without relapse were events of the RFS analysis. Patients who were alive without relapse of BRCA were considered censored. The data for the survival analysis was acquired by univariate and multivariate Cox proportional regression analysis and Kaplan-Meier survival analysis. The comparisons of experimental values between groups were analyzed by Chi-square test, Student’s *t*-test, and one-way ANOVA with post-hoc test. The SPSS (version 20.0, IBM) software was used for the statistical analysis. All the data is expressed as means ± SD from triplicate experiments and representative data is presented. *P* values less than 0.05 were considered statistically significant.

## Abbreviations

95% CI: 95% confidence interval
BCA: breast carcinoma
EMT: epithelial-mesenchymal transition
ER: estrogen receptor
FBS: fetal bovine serum
HER2: human epidermal growth factor receptor 2
HR: hazard ratio
MTT: 3-(4,5-dimethylthiazol -2-yl)-2,5-diphenyltetrazonium bromide
OS: overall survival
PR: progesterone receptor
RFS: relapse-free survival
